# Resolving unknown nucleotides in the IPD-IMGT/HLA database by extended and full-length sequencing of HLA class I and II alleles

**DOI:** 10.1007/s00251-024-01333-z

**Published:** 2024-02-24

**Authors:** Christina E. M. Voorter, Mathijs Groeneweg, Timo I. Olieslagers, Ingrid Fae, Gottfried F. Fischer, Marco Andreani, Maria Troiano, Blanka Vidan-Jeras, Sendi Montanic, Bouke G. Hepkema, Laura B. Bungener, Marcel G. J. Tilanus, Lotte Wieten

**Affiliations:** 1 Department of Transplantation Immunology, Maastricht University Medical Center, P.O. Box 5800, 6202 AZ Maastricht, The Netherlands; 2 GROW School for Oncology and Reproduction, Maastricht University, Maastricht, The Netherlands; 3 Department for Transfusion Medicine and Cell Therapy, Medical University Vienna, Vienna, Austria; 4 Laboratorio di Immunogenetica dei Trapianti, Dipartimento di Oncoematologia, Ospedale Pediatrico Bambino Gesù, Rome, Italy; 5 Tissue Typing Center, Blood Transfusion Centre of Slovenia, Ljubljana, Slovenia; 6 Transplantation Immunology, Department of Laboratory Medicine, University Medical Center Groningen, University of Groningen, Groningen, The Netherlands

**Keywords:** Human leucocyte antigen, Full-length sequencing, NGS, Extended sequences, Group-specific Sanger sequencing

## Abstract

In the past, identification of HLA alleles was limited to sequencing the region of the gene coding for the peptide binding groove, resulting in a lack of sequence information in the HLA database, challenging HLA allele assignment software programs. We investigated full-length sequences of 19 HLA class I and 7 HLA class II alleles, and we extended another 47 HLA class I alleles with sequences of 5′ and 3′ UTR regions that were all not yet available in the IPD-IMGT/HLA database. We resolved 8638 unknown nucleotides in the coding sequence of HLA class I and 2139 of HLA class II. Furthermore, with full-length sequencing of the 26 alleles, more than 90 kb of sequence information was added to the non-coding sequences, whereas extension of the 47 alleles resulted in the addition of 5.5 kb unknown nucleotides to the 5′ UTR and > 31.7 kb to the 3′ UTR region. With this information, some interesting features were observed, like possible recombination events and lineage evolutionary origins. The continuing increase in the availability of full-length sequences in the HLA database will enable the identification of the evolutionary origin and will help the community to improve the alignment and assignment accuracy of HLA alleles.

**Supplementary Information:**

The online version contains supplementary material available at 10.1007/s00251-024-01333-z.

## Introduction

The Human Leucocyte Antigens (HLA) have been widely studied for their role in different immunological processes. It is the most polymorphic gene system described in the human genome, with currently 37,516 different alleles identified in the IPD-IMGT/HLA database (release 3.54, October 2023) (Barker et al. 2023). Many efforts have been taken to elucidate the sequences of individual alleles both for matching purposes in transplantation settings as well as for disease associations and medication hypersensitivity reactions. Historically, this started in the 90 s with Sanger sequencing of PCR amplified cDNA, sequencing the exons coding for the peptide binding groove, also called the antigen recognition domain (Santamaria et al. 1992, 1993). For HLA class I, the peptide binding groove is encoded by exons 2 and 3, whereas for HLA class II, it is encoded by exon 2. With the increase in HLA alleles identified, there was a concomitant increase in ambiguities using the generic PCR amplification as the start for the subsequent sequencing reaction. Therefore, high-resolution workflows often started with either low-resolution typing by sequence-specific oligonucleotides (SSO) or sequence-specific primers (SSP) or ambiguous typing by sequencing to be followed by separate amplification of the two alleles and Sanger sequencing of the separated alleles, which was still limited to the exons encoding the peptide binding groove at that time. Later on, we in our group were able to set up a hemizygous Sanger sequence-based typing (SSBT) approach, enabling full-length sequencing in a group-specific way, resolving all genotype and allele ambiguities (Voorter et al. 2014, 2016). In more recent years, the evolution of next-generation sequencing (NGS) to a more easy-to-perform, more standardized, and more affordable sequencing strategy has paved the way to use this method for HLA typing purposes. Therefore, in many HLA laboratories, routine HLA typing has evolved to full-length sequencing with NGS methods, although complete full-length sequencing of some HLA class II genes is still in development due to the extremely long intron 1 sequences and the presence of both homopolymers and repeat sequences in the introns. These latter two features are still an issue in next-generation sequencing interfering with the correct analysis of such regions.

One of the major goals of the 17th IHIWS in 2017 was to gain and collect full genotyping data of the classical HLA class I and II genes through the application of NGS. According to Albrecht et al. (2017), in the IPD-IMGT/HLA database vs 3.27 from January 2017, the percentage of fully characterized HLA alleles was ranging from 2% for HLA-DRB1 to 16.5% for HLA-C. When retrieving and calculating this information from the IPD-IMGT/HLA database (Robinson et al. 2015), including also HLA-DRB3/4/5, -DQA1 and -DPA1, it ranged from 5% for DRB3/4/5 to 80% for DQA1. In this 3.27 release, the overall coverage for HLA-A, -B, -C, -DRB1/3/4/5, -DQA1, -DQB1, -DPA1, and -DPB1 was 15% fully characterized and 85% only partially known. In the database vs 3.50 from October 2022, the overall coverage was 52% fully characterized and 48% only partially known, with the notice that DRB3/4/5 is the most lagging with 90% of incomplete alleles, probably due to the unavailability of full-length NGS kits or these loci are not the focus of many labs. This successful increase of full-length sequences was the merit of the 17th IHIWS, of a number of HLA laboratories that put effort in providing full-length sequence contributions to the database, and of the incorporation of new technologies that enable routine sequencing of full-length HLA class I genes that are only ~3500 bp in length (Robinson et al. 2020).

In this study, we have again resolved the complete sequences of several alleles that are still incomplete in the database. We have not only performed full-length sequencing for HLA class I, but also for the HLA class II genes DQA1, DQB1, and DPA1. Furthermore, after implementation of NGS full-length sequencing with Illumina for HLA class I a couple of years ago, we noticed that even for some full-length sequenced alleles, there are parts of the gene that could not be aligned to the allele sequence, because these parts, i.e., 5′ UTR and 3′ UTR, are still unknown in the database. Therefore, we have also elucidated these parts and submitted them to the database.

## Materials and methods

Genomic DNA was isolated from diagnostic samples with different methods by the different participants as previously described (Voorter et al. 2018). These diagnostic samples included organ transplantation patients, family members of organ and stem cell transplantation patients, external proficiency testing samples, and samples investigated for disease association or drug hypersensitivity. The HLA alleles included in this report were previously typed by the laboratory of origin, based minimally upon the sequence of the exons encoding the peptide binding groove.

HLA class I full-length sequencing was obtained by our in-house method of group-specific amplification followed by forward and reversed Sanger sequencing according to our SSBT method as previously described (Voorter et al. 2014, 2016). Sequencing analysis was performed with the SeqPilot software (JSI medical systems, Kippenheim, Germany) to obtain the allele call. Since this software can only analyze exons, we have used the Lasergene software (DNA Star, Madison, WI, USA) to analyze the full-length sequences. No separate sequencing and analysis of coding sequences and introns was performed. Confirmation of the full-length sequences was performed by NGS using the AllType Fastplex kit (One Lambda, Life Technologies, Carlsbad, CA, USA) in combination with Illumina sequencing*.* For analysis, the Typestream software (One Lambda) was used for alignment and assignment of exons and introns.

For the HLA class II genes, DQA1, DQB1, and DPA1 full-length sequencing was performed by Illumina NGS using the AllType Fastplex kit and analysis by the Typestream software (One Lambda). Confirmation was performed by a different method based on a second amplification and sequencing of each sample using either the GenDx 3 × 11 kit with Illumina and analysis by the NGSEngine software (GenDx) or MinION sequencing and analysis by both NGSEngine and SeqPilot.

For all Illumina sequencing, the MiSeq System was used in conjunction with micro flow cells. For MinION sequencing, the Flongle R9.4 flow cells were used and the SQK-LSK109 kit for library preparation; a minimum read-depth of > 500 was obtained, far exceeding the reported minimum read-depth of 20 for HLA genotyping by Flongle for deceased donor organ allocation (De Santis et al. 2020). For all full-length sequences, the final alignment of all sequences from one sample was obtained in Lasergene comparing it with an adequate reference allele.

Elucidation of the 5′ and 3′ UTR sequences of already known alleles was performed by NGS using the AllType Fastplex kit in combination with Illumina sequencing. Confirmation was performed by repeating PCR and sequencing as required for IPD-IMGT/HLA database submission.

All extended sequences have been submitted to the EMBL-ENA database and the IPD-IMGT/HLA database. For HLA class I, this was performed using the Typeloader software (Surendranath et al. 2017); for HLA class II, this was done manually. Both the EMBL-ENA accession numbers as well as the obtained IPD-IMGT/HLA submission numbers are indicated in Tables 1 and 5.

**Table 1:** Extended full-length sequenced HLA class I alleles (Table 1, part A) and class II alleles (Table 1, part B) with the EMBL-ENA accession numbers, IPD-IMGT/HLA submission numbers and the number of coding and non-coding nucleotides added to the database

HLA allele	Compared to	EMBL-ENA accession number	IPD-IMGT/HLA submission number	Coding nucleotides added	Non-coding nucleotides added
Part A					
A*03:13	A*03:01:01:01	OW739111	HWS10061654	552^a^	2402
A*03:182 #	A*03:01:01:01	OW739112	HWS10061658	86^b^	2402
A*24:121	A*24:02:01:01	OW739113	HWS10061660	552^a^	2379
A*33:03:07	A*33:01:01:01	OW739110 OX424589	HWS10061648 HWS10065531	552^a^	2419
B*07:23	B*07:02:01:01	OW738604	HWS10061722	543^a^	2670
B*07:24	B*07:02:01:01	OW738597 OX424582	HWS10061662 HWS10065533	543^a^	2894
B*08:01:05	B*08:01:01:01	OW738598	HWS10061664	267^c^	1763
B*15:107	B*15:01:01:01	OW738599	HWS10061666	543^a^	2865
B*18:34 #	B*18:01:01:01	OW738606 OX421043	HWS10061724 HWS10065633	543^a^	3750
B*27:23	B*27:01	OW738600 OX424587	HWS10061684 HWS10065535	543^a^	2953
B*40:38	B*40:01:02:01	OW738601 OX424581	HWS10061680 HWS10065635	543^a^	2894
B*44:13	B*44:02:01:01	OW738602	HWS10061686	0	2223
B*44:22	B*44:02:01:01	OW738603 OX424584	HWS10061690 HWS10065549	543^a^	2894
B*44:90	B*44:02:01:01	OW738605	HWS10061720	543^a^	2214
B*51:136	B*51:01:01:01	OW738607	HWS10061738	77^d^	2897
B*53:06 #	B*53:01:01:01	OX421017	HWS10065637	543^a^	2765
C*03:04:19 #	C*03:04:01:01	OW738943 OX424586	HWS10061742 HWS10065585	555^a^	3102
C*06:11	C*06:02:01:01	OW738945	HWS10061748	555^a^	3079
C*16:201 #	C*16:01:01:01	OW738944 OX424585	HWS10061744 HWS10065605	555^a^	3017
Part B					
DQA1*01:06 #	DQA1*01:01:01:01	OW849871	HWS10061752	519^e^	4915
DQA1*04:04 #	DQA1*04:01:01:01	OW849870	HWS10061754	240^f^	5024
DQB1*03:114 #	DQB1*03:01:01:01	OW849941	HWS10061756	234^ g^	6242
DQB1*06:03:11 #	DQB1*06:01:01:01	OW849942	HWS10061768	516^ h^	6035
DQB1*06:73 #	DQB1*06:01:01:01	OW849944	HWS10061770	516^ h^	6120
DQB1*06:286 #	DQB1*06:01:01:01	OW849943	HWS10061782	14^i^	6125
DPA1*01:03:14 #	DPA1*01:03:01:01	OW849872	HWS10061750	100^j^	4706

In some cases, the 5′ and 3′ UTR sequences were separately submitted, revealing two different submission numbers in these cases. All alleles were compared with the first allele of the same allele group; a hash symbol behind the allele indicates that differences were found between the non-coding (5′ UTR/introns/3′ UTR) sequences of the full-length sequenced HLA allele and the reference allele of comparison

^a^Coding sequence of only exons 2 and 3 are known, addition of all other exons (1, 4, 5, 6, 7, 8 for HLA-A and -C; 1, 4, 5, 6, 7 for HLA-B)

^b^Exons 6, 7, and 8 added

^c^Exons 1, 5, 6, and 7 added

^d^Exons 6 and 7 added

^e^Exons 1, 3, and 4 added

^f^Exons 1 and 4 added

^g^Exons 1, 4, 5, and 6 added

^h^Exons 1, 3, 4, 5, and 6 added

^i^Exon 6 added

^j^Exon 1 added

Analysis of sequences was performed as previously described (Voorter et al. 2018): the obtained genomic sequences were compared with the genomic sequence of the first allele of the same allele group with the lowest number of allele name for which a genomic sequence was known (IPD-IMGT/HLA database, release 3.51) as indicated in Tables 1 and 5. Only differences in the intron sequences (for Table 1) or in the extended 5′ and 3′ part (for Table 5) were considered. When differences were observed, the genomic sequence of the allele was compared to other alleles of the same allele group to determine whether the intron and/or 5′ and 3′ UTR sequences were already known for that allele group. If the sequence was not yet known for the allele group, the sequence was compared to the genomic sequences of all other allele groups to enable identification of previously conserved and variable positions and possible recombination or gene conversion events between different allele groups. Positions correspond to the genomic position obtained when full-length sequences with the reference allele were compared in IPD-IMGT/HLA database.

Phylogenetic trees were based on ClustalW multiple alignment. Building was done using the free software R 4.2.2 (https://r-project.org) with the seqinr 4.2-23 (https://cran.r-project.org/web/packages/seqinr/), ape 5.7-1 (https://cran.r-project.org/web/packages/ape/), and msa 1.30.1 (https://www.bioconductor.org/packages/release/bioc/html/msa.html) libraries and their dependencies (Bodenhofer et al. 2015; Charif and Lobry 2007; Paradis and Schliep 2019). For the analysis, only alleles that had known sequences in the analyzed region (see figure legends) were used. Alignments of the different alleles were performed with the ClustalW algorithm from the msa package (Thompson et al. 1994). Distances between the tree nodes were calculated as the square root of the percentage difference between each individual sequence used (Charif and Lobry 2007).

## Results

### Full-length sequencing of incomplete alleles

The sequences of 19 HLA class I (Table 1, part A) and 7 HLA class II (Table 1, part B) alleles were extended to full-length sequences. In most cases, only the sequences of exons 2 and 3 for HLA class I and exon 2 for HLA class II were present in the IPD-IMGT/HLA database; there was no information on the non-coding sequence available for any of these alleles. For HLA class I, we resolved 8638 unknown nucleotides for the coding sequence and 51,582 for the non-coding sequence; for HLA class II, we resolved 2139 unknown nucleotides for the coding and 39,167 for the non-coding region.

### HLA class I

Coding nucleotides could be added to the database for 18 out of the 19 HLA class I alleles, because of previously unknown exon sequences (Table 1, part A). Only for B*44:13, no coding nucleotides could be added because the exon sequences were already known. For each allele, the number of coding and non-coding nucleotides that could be added to the database is indicated in Table 1, part A. In 14 out of 19 alleles, no differences with the reference alleles were found in the non-coding regions. In 5 out of 19 alleles, there was a difference in the non-coding (5′ UTR/introns/3′ UTR) regions of the allele compared to the first allele of the same allele group (indicated in Table 1A by a hash symbol). These differences are listed in Table 2. More detailed evaluation of possible recombinations showed the following:

**Table 2:** Alleles with differences in the non-coding (5′ UTR/introns/3′ UTR) sequences compared to the reference allele

HLA allele	Compared to	Differences in non-coding regions	Comment
A*03:182	A*03:01:01:01	I6 C2605T	Not unique for A*03
B*18:34	B*18:01:01:01	I3 T1127C, I5 A2180G, 3' UTR T3014C, C3358T, C3472T, C3609T	No differences with B*18:01:01:02
B*53:06	B*53:01:01:01	10 differences^a^	Possible recombination
C*03:04:19	C*03:04:01:01	I3 G1030A	Possible recombination
C*16:201	C*16:01:01:01	5’ UTR G-415A	Unique for C*16
DQA1*01:06	DQA1*01:01:01:01	Many differences^b^	Possible recombination
DQA1*04:04	DQA1*04:01:01:01	3205 Ins A	homopolymer
DQB1*03:114	DQB1*03:01:01:01	I1 C744T, I2 G3363A	Not unique for DQB1*03
DQB1*06:03:11	DQB1*06:01:01:01	> 300 differences	No differences with DQB1*06:03:01:01
DQB1*06:73	DQB1*06:01:01:01	> 300 differences	No differences with DQB1*06:02:01:01
DQB1*06:286	DQB1*06:01:01:01	> 300 differences	No differences with DQB1*06:02:01:01
DPA1*01:03:14	DPA1*01:03:01:01	I1 G1723A, I1 C3318G, I2 G4149C	Not unique for DPA1*01

*I* intron, *E* exon, *UTR* untranslated region, number indicates the position of the genomic sequence according to the IPD-IMGT/HLA database

^a^See Table 3 for details

^b^See Table 4 for details

By comparing B*53:06 with B*53:01:01:01, a total of 10 differences were found: 1 in exon 2, 1 in intron 2, 2 in exon 3, and 6 in the 3′ UTR region (Table 3). In fact, these differences were not found in any of the other B*53 alleles, and therefore the sequence was compared to all other HLA-B alleles in the IPD-IMGT/HLA database. It turned out that the sequence of B*53:06 is identical to B*51:01:01:04 up to genomic position 726 and from 810 to the end of the 3′ UTR. The part between 695 and 900 is identical to B*53:01:01:01 and many other B*53 alleles. Therefore, the B*53:06 allele may have arisen by a gene conversion event with a double cross over, resulting in a recombination of B*51:01:01:04 with a B*53 allele, exchanging the first part of exon 3, with breakpoints between 695–726 and 810–900 (Table 3). With the first identification of this allele, it was already serological typed as B53/B51-like variant (Anholts et al. 2001), the expert assigned type was also B53/B51 (Holdsworth et al. 2009), the neural network assignment was B53 (Maiers et al. 2003), and also the recently published systematic classification of serological specificities assigned a B53 serotype, with the comment being short cross reactive (Osoegawa et al. 2022).

**Table 3:**
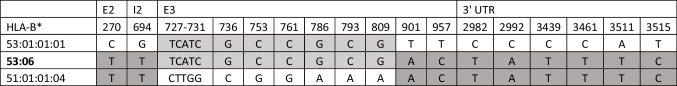
Comparison of part of the sequences of B*53:01:01:01, B*53:06, and B*51:01:01:04 illustrating that B*53:06 may have arisen by a gene conversion event with double cross over, recombining B*51:01:01:04 and a B*53 allele

Heterozygous positions that are identical for B*53:01:01:01 and B*53:06 are in light grey, heterozygous positions identical for B*51:01:01:04 and B*53:06 are in dark grey

*I* intron, *E* exon, *UTR* untranslated region, number indicates the position of the genomic sequence according to the IPD-IMGT/HLA database

Comparing the full-length sequence (including exons) of C*03:04:19 with C*03:04:01:01 revealed that there are only 2 nucleotide differences, one in exon 3 at position 993 (G > A) and one in intron 3 at position 1030 (G > A). Since both A’s are present in most C*07 alleles, it could be possible that C*03:04:19 was the result of a recombination between C*03:04 and a C*07 exchanging a part between 970 and 1080 of C*07 into the C*03:04 allele. Except for the majority of the C*07 alleles, the presence of both A’s (993 and 1030) together was only detected in 2 other C alleles, C*12:181 and C*15:02:33:01. It is tempting to speculate that these alleles also arose in the same way.

For B*27:23, we did not observe any differences with B*27:01 in non-coding sequences (Table 1, part A). But this latter sequence is only known till position 2707, 30 nucleotides after the stop codon. Therefore, the remaining nucleotides of the 3′ UTR region from B*27:23 were also compared with B*27:04:01 and B*27:05:02:01, the two sequences that are known till the end at position 3799. Compared with B*27:05:02:01, the B*27:23 allele shows no differences in 3′ UTR, whereas there are 4 differences with B*27:04:01 (T2812C, T3474C, C3574T, T3611C). At the first discovery of this allele, it was already noticed that this allele arose by a gene conversion event between B*27:05:02 and a B*35 allele (Darke et al. 2002).

### HLA class II

In all HLA class II allele cases, both coding and non-coding nucleotides could be added to the database (Table 1, part B); in all cases, no non-coding sequence was available. In all alleles, there was a difference in the non-coding (5′ UTR/introns/3′ UTR) regions of the allele compared to the first allele of the same allele group, and these differences are listed in Table 2. Details about some interesting observations are as follows:

Comparing DQA1*01:06 with DQA1*01:01:01:01 revealed a striking phenomenon for this DQA1*01:06 allele. Whereas the 5′ and 3′ UTR sequences of DQA1*01:06 are identical to DQA1*01:01:01:01, there are many differences between these alleles in the intron sequences (Table 4). In fact, all intron sequences of DQA1*01:06 are identical to DQA1*01:02:01:04/14, but there are differences in the 5′ and 3′ UTR between these alleles. Concerning the exon sequences, DQA1*01:06 is identical to DQA1*01:02:01:04/14 except for one nucleotide in exon 2 at position 3974 (gDNA, = position 199 cDNA, codon 44), which has been reported as a unique mutation for DQA1*01:06 at the first discovery of this allele (Luo et al. 1999), and still is; no other DQA1 allele has a G at this position; in all alleles, an A nucleotide is conserved. All together, this suggests that DQA1*01:06 is a unique allele that may have arisen by a double cross over event between DQA1*01:01:01:01 and one of the alleles DQA1*01:02:01:04 or DQA1*01:02:01:14 with an additional point mutation at position 3974 (Table 4).

**Table 4:**
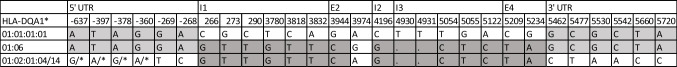
Comparison of part of the sequences of DQA1*01:01:01:01, DQA1*01:06, and DQA1*01:02:01:04/14 illustrating the DQA1*01:06 may have arisen by a gene conversion event with double cross over, recombining DQA1*01:01:01:01 and DQA1*01:02:01:04/14, and an additional mutation at position 3974

Heterozygous positions that are identical for DQA1*01:01:01:01 and DQA1*01:06 are in light grey, heterozygous positions identical for DQA1*01:02:01:04/14 and DQA1*01:06 are in dark grey

For intron 1, only the first and last 3 heterozygous positions are shown

*I* intron, *E* exon, *UTR* untranslated region, number indicates the position of the genomic sequence according to the IPD-IMGT/HLA database, “.” = deletion, “*” = unknown sequence

Beside a difference in exon 3, the DQA1*04:04 has another difference with DQA1*04:01:01:01, namely an insertion of an A at position 3205, resulting in a homopolymer of 13 A nucleotides compared to 12 in DQA1*04:01:01:01. Of the 22 DQA1*04 alleles for which the intron 1 sequence is known, 5 have a homopolymer of 12A, 13 have a homopolymer of 13A, and 4 have a homopolymer of 14A. All sequencing methods have difficulty with accurately sequencing homopolymers. Although we used 3 different sequencing methods for this part of the DQA1*04:04 allele, the real number of A nucleotides present in this homopolymer is hard to determine, although with all three methods, the different analysis programs identified 13 A nucleotides at this position.

**Fig. 1:**
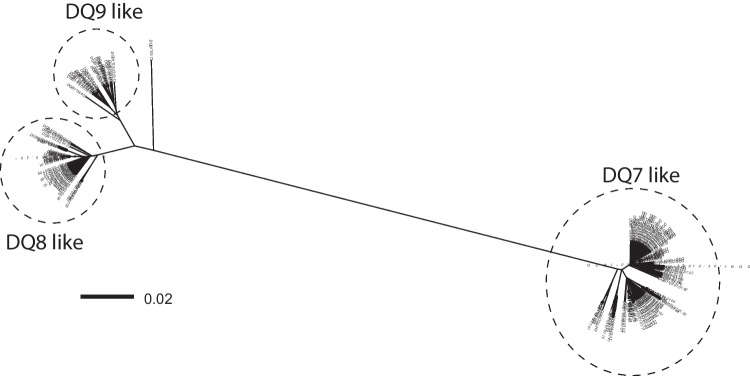
Phylogenetic tree of HLA-DQB1*03. A multiple sequence alignment was performed on the full-length sequences from position −150 up to 6502 without exon 2 from all DQB1*03 alleles for which this sequence was available in the IPD-IMGT/HLA database (vs. 3.51), excluding null and Q alleles. Since Osoegawa et al. (2022) already identified DQB1*03:06 and *03:25* as DQ4 serotypes, we have excluded these alleles from this phylogenetic tree. Details of the alleles are listed in Supplemental Table 1A. The outlier, DQB1*03:72, was serotyped DQ9 by Osoegawa et al. (2022), but the full-length sequence was completely identical to DQB1*04:02:01:04, except exon 2. The scale bar indicates the length of the tree edges, corresponding to the differences between two allele sequences as calculated by the R SeqinR package (Charif and Lobry 2007)

Comparing the full-length sequence of the DQB1*03:114 allele with all other DQB1*03 alleles revealed huge differences in both coding and non-coding nucleotide sequences of the alleles belonging to the serological specificity DQ7 compared with DQ8/DQ9 specificity, whereas there are only a few differences between DQ8 and DQ9. The full-length sequence of the allele DQB1*03:114 could be identified as a DQ7 sequence. Since the serological specificity is merely depending on the serological reaction with specific antibodies against the beta-1 domain of the HLA molecule, we have also checked the exon 2 sequence of this allele. Osoegawa et al. (2022) have determined that the critical residues for DQ7/8/9 are amino acids 45, 57, 74, 84, and 85, the latter 3 are identical for all 3 types, but important for distinguishing them from the other serological DQ types. In fact, the difference between DQ7, DQ8, and DQ9 only depends on the amino acids 45 and 57, where ED or EA encodes DQ7, GA encodes DQ8 and GD DQ9. The allele DQB1*03:114 has ED at these positions, clearly identifying this allele as a DQ7 serotype, which fits with the full-length sequence. To investigate whether these full-length sequence differences always fit with the serological type, we prepared a phylogenetic tree, based on the full-length sequences (−150–6502) of the DQB1*03 alleles available in the IPD-IMGT/HLA database without exon 2, depicted in Fig. 1. This phylogenetic tree shows three distinct groups, one reflecting the DQ7 like sequence group, one DQ8 like, and one DQ9 like group, with less distance between DQ8 and DQ9, because of less differences. Comparing the alleles in these groups with their final serological assignment based on the two amino acids in exon 2, as compiled by Osoegawa et al. (2022) (Supplemental Table 5 of their paper), revealed 2 alleles, where the serological type does not fit with the full-length sequence type, namely DQB1*03:10 and *03:12*, both serologically typed as DQ9 according to Osoegawa et al. (2022), whereas their full-length sequence is DQ7 like. DQB1*03:10 was previously identified by the experts as DQ3, by the neural network as DQ7 (Maiers et al. 2003), and assigned by the WHO as DQ8 (Barker et al. 2023), whereas DQB1*03:12 was also by the experts and by the neural network identified as DQ9. Both alleles have sequences completely identical to DQB1*03:01:01 except for exon 2, with 1 (DQB1*03:10:01), 2 (DQB1*03:10:02), or 4 (DQB1*03:12) nucleotide differences, changing the crucial amino acid 45 from E to G. Whether these alleles evolved from a double recombination between DQB1*03:01:01 and DQB1*03:03 or whether they arose by point mutation(s) in exon 2 is not clear.

**Fig. 2:**
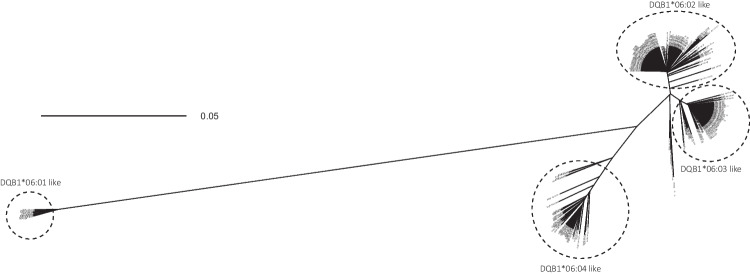
Phylogenetic tree of HLA-DQB1*06. A multiple sequence alignment was performed on the full-length sequences from position −30 up to 6410 from all DQB1*06 alleles for which this sequence was available in the IPD-IMGT/HLA database (vs. 3.51). Details of the alleles are listed in Supplemental Table 1B. The scale bar indicates the length of the tree edges, corresponding to the differences between two allele sequences as calculated by the R SeqinR package (Charif and Lobry 2007)

Comparing the DQB1*06 full-length sequences of the three alleles in this study, DQB1*06:03:11, DQB1*06:73, and DQB1*06:286 with the first allele of this allele group, DQB1*06:01:01:01, we noticed that the DQB1*06:01 alleles showed huge differences with all other DQB1*06 alleles in both the coding and non-coding part, except *06:103* and *06:243*, implicating that these alleles belong to one lineage within the DQB1*06. Also, between other DQB1*06 groups, differences were observed in both coding and non-coding regions, but to a lesser extent. To determine the lineages within the DQB1*06 allele group, we prepared a phylogenetic tree, shown in Fig. 2, using the full-length sequences (−30–6410) of all available DQB1*06 alleles in the IPD-IMGT/HLA database. As expected, there was one lineage which had a huge evolutionary distance to the other lineages and was composed of all 8 DQB1*06:01 alleles, DQB1*06:103 and DQB1*06:243. The other DQB1*06 alleles could be divided in 3 further lineages, DQB1*06:02like, composed of DQB1*06:02 and similar alleles, DQB1*06:03like and DQB1*06:04like, to which also the more frequent DQB1*06:09 alleles belonged. This tree also shows that there are not so many differences between DQB1*06:02like and *06:03*like as between DQB1*06:02like and *06:04*like or between *06:03*like and *06:04*like. This information might be helpful in regard to matching possibilities and antibody definition.

### Addition of 5′ and 3′ UTR sequences of HLA class I

During full-length sequencing for diagnostic purposes, we noticed that some full-length sequenced alleles lacked parts of the 5′ and/or 3′ UTR sequence. Table 5 shows the 47 alleles for which we added additional 5′ and/or 3′ UTR sequences to the already existing allele sequence. In total, we added 5.5 kb unknown nucleotides to the 5′ UTR sequence of 24 alleles and > 31.7 kb to the 3′ UTR sequence of 47 alleles. In none of the cases did comparison of these sequences with the reference allele reveal a difference in the 5′ UTR region. But, in 24 of the cases, differences were found in the 3′ UTR region (Table 5).

**Table 5:** HLA class I alleles extended with 5′ and 3′ UTR sequences with the EMBL-ENA accession numbers, IPD-IMGT/HLA submission numbers and the number of non-coding nucleotides for 5′ and 3′ UTR added to the database

HLA allele	Compared to	EMBL-ENA accession number	IPD-IMGT/HLA submission number	Non-coding nucleotides added 5′ UTR	Non-coding nucleotides added 3′ UTR	Differences in extended sequence
A*02:01:01:48	A*02:01:01:01	OX421037	HWS10065285	0	185	
A*29:95	A*29:01:01:01	OX421046	HWS10065287	150	231	
B*07:02:01:32	B*07:02:01:01	OX421012	HWS10065289	248	1022	
B*07:06:01:01	B*07:02:01:01	OX463247	HWS10065965	0	659	T3358C, C3698G
B*07:436	B*07:02:01:01	OX421031	HWS10065291	0	659	C3053T, C3129T, G3341A, T3358C, T3472C, T3576C, A3636G
B*08:09	B*08:01:01:01	OX421023	HWS10065293	0	660	
B*14:81	B*14:02:01:01#	OX421015	HWS10065295	248	986	
B*15:03:01:01	B*15:01:01:01	OX421050	HWS10065297	284	1022	C2902T, C3174T, T3524C, G3542A, C3622T, C3681A
B*15:07:01:02	B*15:01:01:01	OX421032	HWS10065299	16	766	
B*15:13:01	B*15:01:01:01	OX421014	HWS10065301	16	1011	T3485C, T3524C, C3622T, C3681A
B*15:37	B*15:01:01:01	OX421035	HWS10065303	57	777	T3524C, G3542A, C3622T, C3681A
B*18:01:01:18	B*18:01:01:01	OX421036	HWS10065305	0	659	C3358T
B*18:18:01:02	B*18:01:01:01	OX421052	HWS10065309	0	983	T3014C, C3358T, C3472T, C3576T, C3609T, C3668A
B*18:144	B*18:01:01:01	OX421022	HWS10065307	0	670	C3358T, C3472T, C3576T, C3609T, C3668A
B*27:06:01:01	B*27:04:01#	OX421057	HWS10065311	0	659	T3509A, G3531A
B*35:05:01:01	B*35:01:01:02#	OX421028	HWS10065313	0	129	C3604T, C3611T
B*35:30:01:01	B*35:01:01:02#	OX421047	HWS10065315	0	984	A3188C, T3255G, A3259G, G3317A, T3362C, G3363A, T3377G, G3381T, T3389C, G3416C, T3439C, T3461C, C3604T, C3611T
B*40:23	B*40:01:02:01#	OX421021	HWS10065537	0	659	
B*40:27:01	B*40:02:01:01	OX421054	HWS10065539	0	661	
B*41:02:01:05	B*42:01:01:01#^a^	OX421040	HWS10065541	0	1005	
B*44:02:01:14	B*44:02:01:01	OX421033	HWS10065543	0	659	
B*44:03:01:14	B*44:02:01:01	OX421055	HWS10065545	0	660	T3412G, T3472C, T3506A, G3523A
B*44:04	B*44:02:01:01	OX421048	HWS10065547	16	659	T3412G, T3472C, T3506A, G3523A
B*49:01:01:02	B*49:01:01:01	OX421056	HWS10065551	0	659	
B*51:01:01:14	B*51:01:01:01	OX421020	HWS10065553	131	811	T3525C, G3702C
B*51:01:01:25	B*51:01:01:01	OX421034	HWS10065555	27	679	G3702C
B*51:01:01:31	B*51:01:01:01	OX421045	HWS10065557	0	699	C3439T, C3461T, C3613T, G3702C
B*56:01:01:03	B*56:01:01:15#	OX463248	HWS10065969	0	659	
B*81:01:01:04	B*81:01:01:01^b^	OX421058	HWS10065559	248	1022	
C*02:02:02:06	C*02:02:02:01	OX421019	HWS10065561	0	235	
C*02:02:02:14	C*02:02:02:01	OX421044	HWS10065569	192	638	
C*02:02:02:20	C*02:02:02:01	OX421016	HWS10065571	212	638	
C*02:02:02:27	C*02:02:02:01	OX421027	HWS10065573	431	797	
C*02:06:01	C*02:02:02:01	OX421038	HWS10065575	201	652	
C*03:02:02:02	C*03:02:02:01#^c^	OX421051	HWS10065577	212	589	A3196G, G3200C, C3305T, G3325T, A3372C, 3387_3388insG, C3423T, G3429A, C3439T, G3498A
C*03:03:01:22	C*03:02:02:01#^c^	OX421018	HWS10065581	431	801	A3196G, G3200C, C3305T, G3325T, A3372C, 3387_3388insG, C3423T, G3429A, C3439T, G3498A
C*03:04:02:01	C*03:02:02:01#^c^	OX421042	HWS10065583	0	278	A3196G, G3200C, C3305T, G3325T, A3372C, 3387_3388insG, C3423T, G3429A, C3439T, G3498A
C*04:01:01:19	C*04:01:01:06#	OX421049	HWS10065587	111	620	
C*04:01:01:38	C*04:01:01:06#	OX421029	HWS10065589	431	820	
C*06:02:01:21	C*06:02:01:01	OX421024	HWS10065591	431	819	G3033C
C*07:02:01:44	C*07:01:01:01	OX421030	HWS10065593	435	819	G3000A, T3001C
C*07:46	C*07:01:01:01	OX421053	HWS10065595	204	658	
C*12:03:01:28	C*12:02:02:01	OX421039	HWS10065597	431	794	C3638T, G3664A
C*12:143	C*12:02:02:01	OX421025	HWS10065599	0	291	C3638T, G3664A
C*12:167	C*12:02:02:01	OX421013	HWS10065601	373	794	G3664A
C*15:06:01:01	C*15:02:01:01	OX421026	HWS10065603	0	291	
C*17:01:01:05	C*17:01:01:02	OX421041	HWS10065607	0	286	

The extended sequences were compared with the first allele of the same allele group, and the differences are depicted in the last column. A hash symbol behind the reference allele indicates that this is not the first allele of the allele group for which the genomic sequence is known, because the first allele is missing part of the 3′ UTR region

^a^This B*41:02:01:05 allele had to be compared with another allele group, because there was no B*41 in the database with known sequence up to position 3698

^b^The last 131 bp of this B*81:01:01:04 allele could not be compared to any B*81 allele, because it was unknown for all B*81 alleles

^c^The allele C*03:02:02:01 was the first allele of the C*03 allele group of which the full-length sequence is known, but this allele together with 03:544 and 03:614 showed these 10 differences with the alleles studied, all other C*03 alleles showed sequences identical to the 3 studied alleles

In some cases, the sequence could not be completely compared with the first allele of the allele group, because of the missing part of the 3′ UTR sequence. In those cases, the next allele from the allele group with sufficient sequence information was chosen as reference sequence, indicated with a hash symbol in Table 5.

In most cases, the differences in the resolved 3′ UTR region were also observed in other alleles of the same allele group. Some interesting cases are described in more detail here.

B*07:436 showed, compared to B*07:02:01:01, 7 nucleotide differences in the 3′ UTR part between position 3039 and 3698 that we added to the already existing part. The 3′ UTR sequence of this B*07:436 was rather different from any B*07 allele, and comparison with other B alleles showed that this sequence was identical to many of the B*37 alleles. The B*07:436 allele was previously known as B*07:02:06, because there were only 3 silent substitutions found in exon 4 compared with B*07:02:01:01 (Anholts et al. 2009). Sequencing of the other exons revealed non-silent differences in exons 5 and 7, and therefore the allele was renamed in 2021 (Barker et al. 2023). In fact, comparison of the full-length sequences of B*07:02:01:01, B*07:436, and all B*37 alleles clearly shows that the 5′ end of B*07:436 is identical to B*07:02:01:01 up to position 1526, whereas the 3′ end is identical to many B*37 alleles up to position 1411, suggesting that this allele B*07:436 is a recombinant between B*07:02:01:01 and one of the B*37 alleles with the break point somewhere between location 1411 and 1526. Since we had offspring of this individual, we were able to determine the haplotype on which B*07:436 was located, being A*01:01:01, B*07:436, C*06:02:01, DRB1*15:01:01, DRB5*01:01:01, DQA1*01:02:01, and DQB1*06:02:01. Concerning the B~C association, the C*06:02 is very common present with B*37 and only rare with B*07 in the European population (Gragert et al. 2013), concordant with a recombination event.

**Fig. 3:**
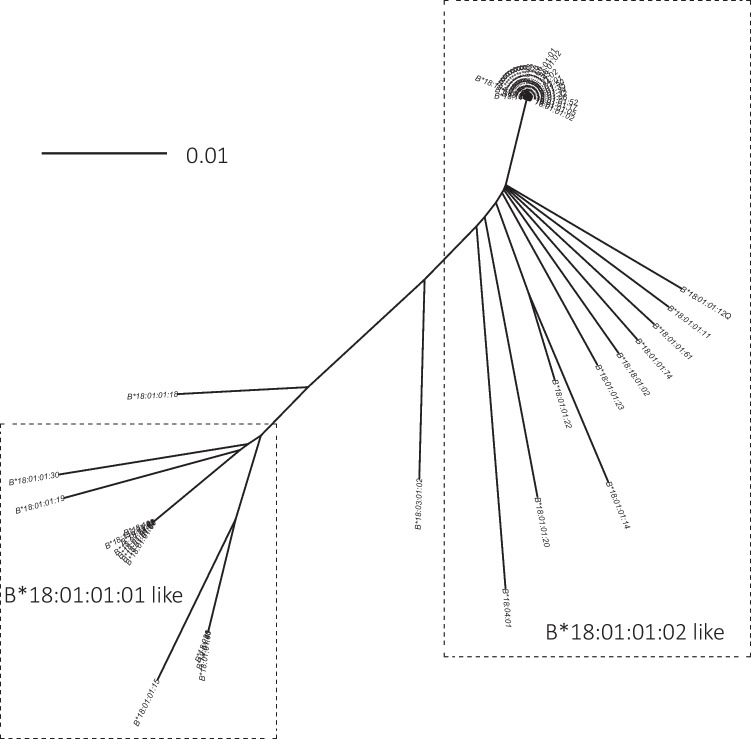
Phylogenetic tree of HLA-B*18. A multiple sequence alignment was performed on the 5′ UTR, intron, and 3′ UTR sequences from position −20 up to 3500 from all B*18 alleles for which these sequences were available in the IPD-IMGT/HLA database (vs. 3.51). Details of the alleles are listed in Supplemental Table 1C. Of the two alleles outside the two clusters, the B*18:03:01:02 has 2 nucleotides identical to B*18:01:01:01 (1127 T, 3358C) and 3 identical to B*18:01:01:02 (2180G, 3014C, 3472 T), and the B*18:01:01:18 allele has 3 nucleotides identical to B*18:01:01:01 (1127 T, 2180A, 3472C) and 2 identical to B*18:01:01:02 (3014C, 3358 T). The scale bar indicates the length of the tree edges, corresponding to the differences between two allele sequences as calculated by the R SeqinR package (Charif and Lobry 2007)

In line with our previous study (Voorter et al. 2018), we observed differences in the introns and 3′ UTR in the B*18 group. The differences are limited to certain positions and are in fact pointing to a lineage evolutionary origin of the B*18. To investigate whether there is indeed a separation into two lineages, we prepared a phylogenetic tree using the sequences available in the IPD-IMGT/HLA database from 5′ UTR, introns, and 3′ UTR from position −20 up to position 3500, including all B*18 alleles of which these sequences are known. Figure 3 shows a clear separation between all B*18:01:01:01 and B*18:01:01:02 like sequences that have differences in intron 3 position 1127 (T/C), intron 5 position 2180 (A/G), and 3′ UTR positions 3014 (T/C), 3358 (C/T), and 3472 (C/T). Positions 3609 (C/T) and 3668 (C/A), which also showed clear association with the two lineages, could not be taken into account, because there are too many alleles of which this sequence part is not known.

## Discussion

In this study, the full-length sequences of 19 different HLA class I and 7 different HLA class II alleles were obtained as well as 5′ and 3′ UTR sequences of 47 class I alleles and submitted to the EMBL-ENA and IPD-IMGT/HLA database. For HLA class II, DQA1, DQB1, and DPA1 are the only genes that can be full-length sequenced with the currently available NGS HLA sequencing kits due to their limited lengths ranging from 7000 to 10,000 bp, and therefore we restricted our analysis to these genes.

Of the 19 HLA class I alleles for which the full-length sequence was resolved in this paper, 14 were noted as well documented in the CIWD3.0.0 dataset (Hurley et al. 2020) in the total population (A*03:13, A*24:121, A*33:03:07, B*07:23, B*07:24, B*08:01:05, B*18:34, B*27:23, B*44:13, B*44:22, B*53:06, C*03:04:19, C*06:11, C*16:201), whereas 12 of them were well documented within Europe. Within the European CWD catalog, where alleles at the 2nd field level were taken into account, only 3 of the 19 class I alleles were described as well documented (B*07:23, B*07:24, B*18:34) (Sanchez-Mazas et al. 2017). The other 5 alleles were not well-documented in CIWD3.0.0, and one of the reasons could be that they belong to a G group that might not have been identified in the stem cell registries used for this CIWD table, due to limited typing resolution. Of the 4 DQB1 alleles, 3 were well documented in CIWD 3.0.0 both in the total population as well as in the European population (DQB1*03:114, DQB1*06:03:11, DQB1*06:73), whereas only one was reported by the European CWD as well documented (DQB1*06:73). For DQA1 and DPA1, no list of CIWD alleles has been provided in the CIWD 3.0.0. In the European CWD (Sanchez-Mazas et al. 2017), there is a list of DQA1 alleles, and in the CWD 2.0.0 (Mack et al. 2013), there is a list of both DQA1 and DPA1, but the alleles from the present study were not reported in either of them. However, Cordovado and co-authors reported their newly identified DQA1*04:04 allele to be present in 5 unrelated European participants (Cordovado et al. 2005).

In our previous publication, we already noticed that there are HLA-B allele groups that show several nucleotide differences in the introns, whereas other HLA-B allele groups are rather conserved (Voorter et al. 2018). In line with this previous study, little intron variation was seen in the B*07, B*27, and B*44 alleles, although there was some variation in the 3′ UTR region. This variation in 3′ UTR may have a biological impact, since 3′ UTR regions can contain binding sites for RNA binding proteins and microRNAs, that can play a role in modulating mRNA stability, localization, and translation efficiency, and thus in fine-tuning of gene expression levels.

Four of the alleles (B*53:06, B*07:436, C*03:04:19, and DQA1*01:06) described here could have originated from different allele groups by intragenic gene conversion, exchanging sequence information as a result of single or double cross over events. Decades ago, recombination by gene conversion and cross over was already recognized as one of the key players in creating the diversity of HLA molecules (Carrington 1999; Högstrand and Böhme 1999; Parham and Ohta 1996; Yeager and Hughes 1999). Since then, many HLA alleles have been determined to originating through recombination events, mostly intragenic (between alleles of the same locus) and rarely intergenic (between alleles of different loci). Among HLA-B alleles, B*53 and B*07 have previously been reported to be involved in recombination events; B*53:31 was identified as arisen by a gene conversion event between B*35:03:01 and B*44:02 or B*13:02 (Adamek et al. 2015), B*07 has been reported as donor allele group for the alleles B*44:150 and B*08:79 (Balas et al. 2012a, b), but also for the allele A*23:31 that originated from an interlocus gene conversion (Lazaro et al. 2012). Several B*07 alleles might also have been the result of a recombination event themselves, like B*07:12, that seems to be a recombination between B*07:02 and either B*35, *53*, or *58*, sharing intron 2 and first part of exon 3 sequences with the latter allele groups (Steiner et al. 2001). Also within the C*03 allele group, intralocus recombination was detected, for example, the C*03:58 allele that turned out to be a hybrid of C*03:03:01 and C*01:03 (Lazaro et al. 2011). For DQA1, most alleles only differ from another allele by a single nucleotide difference, implicating that they result from a single point mutation. However, there are alleles that show more than one nucleotide difference with all known DQA1 alleles, like DQA1*05:21, which might actually be the result of a recombination between DQA1*05:05:01:13 and any DQA1 allele but DQA1*05 (Ingram et al. 2020).

In addition to the repeat regions present in the non-coding sequences of HLA class II genes that cause problems in correct sequence analysis, homopolymers might be difficult to resolve appropriately. During analysis of the DQA1*04:04 sequence, we noticed such a homopolymer in intron 1 of DQA1 starting at position 3105. Comparing all known sequences of DQA1 for this part revealed that all DQA1*02 alleles have 7 A’s in a row and all DQA1*03 and DQA1*05 alleles have 9 A’s, whereas the DQA1*01, *04*, and *06* alleles have a number of A nucleotides varying between 10 and 15. It is remarkable that there is no variation in A repeat length as long as this repeat is 9 nucleotides or less, which might be the limit of what can be reliably analyzed by Sanger sequencing and NGS methods. We have carefully analyzed the DQA1*04:04 allele for this homopolymer region with 3 different sequencing methods, NGS by Illumina, Sanger sequencing, and MinION sequencing. With all 3 methods, the presence of 13 A nucleotides at this position was supported. The question is whether the variability that is observed within this homopolymer for alleles that are identical to each other, but only differ in homopolymer length, truly reflects polymorphism or whether it is due to limitations of the sequencing methods used at that time. It was previously proposed that homopolymer tracts can be expanded via replication slippage, but that this slippage-mediated expansion does not work on tracts with lengths below a critical threshold of 7–10 nucleotides (Dechering et al. 1998). This might be exactly the reason that there is variation in this homopolymer A tract in DQA1*01/04/06, because the homopolymer has 10 or more nucleotides. On the other hand, it can also be the reason that because of this replication slippage, homopolymer tracts of 10 or more nucleotides cannot reliably be sequenced with standard genotyping methods. Although modulation of transcription and/or organization of the chromatin structure has been proposed as a functional role for homopolymer tracts (Brahmachari et al. 1997; Dechering et al. 1998), it is unclear whether the variation in homopolymer length in the introns of the HLA genes has any added value. Within the DQA1*04:01:01, there are 3 sets of alleles DQA1*04:01:01:01/02/03, DQA1*04:01:01:04/05, and DQA1*04:01:01:07/09 that have complete identical sequences, but different homopolymer lengths at this position in intron 1. It would be interesting to see whether these homopolymer length differences really reflect the natural diversity of these HLA alleles and whether they indeed have any impact on their functional properties. It would also be interesting to see when all DQA1*04 alleles would be reduced to identical homopolymer length in the database, whether the HLA assignment algorithms for NGS methods will identify different polymer lengths at all, if they are not in the database as reference sequences. HLA typing could be more simplified if there is no need to resolve both homopolymers and short tandem repeat regions present within the HLA alleles. A possibility would be to implement an annotation on these positions to indicate the presence of such a difficult region.

In summary, we have investigated the full-length sequences of some HLA class I and HLA class II alleles that were not yet available in the IPD-IMGT/HLA database, providing a small but worthy contribution to this database. In the latest version of the IPD-IMGT/HLA database (v 3.54.0, Oct 2023), the overall coverage was 54% full characterized (including 2.5% cDNA only) and 46% only partially known. With the efforts of international workshops, international donor registries and other HLA laboratories with routine sequencing facilities the gaps in the IPD-IMGT/HLA database are filled more and more. This information on full-length HLA sequences can contribute to knowledge about the evolutionary origin of the allele and can facilitate the recognition of different lineages within a certain group of alleles. Furthermore, filling the gaps in the IPD-IMGT/HLA database will help the community to get better alignment and assignment of alleles and improve the accuracy of HLA typing.

### Supplementary information

Below is the link to the electronic supplementary material.

Supplementary file1 (DOCX 18 KB)
