# Supplementary material for: Resolving unknown nucleotides in the IPD-IMGT/HLA database by extended and full-length sequencing of HLA class I and II alleles
Source: Immunogenetics. 2024 Feb 24;76(2):109–21. doi: 10.1007/s00251-024-01333-z (PMC10944811; doi:10.1007/s00251-024-01333-z)
Supplement: Supplementary file 1 — Supplementary file1 (DOCX 18 KB) [file 251_2024_1333_MOESM1_ESM.docx]

SUPPLEMENTAL TABLE 1: Allele names depicted in figures 1-3

TABLE 1A: Allele names from figure 1 divided in the clusters DQ7like, DQ8like and DQ9like. The alleles not falling within these clusters are indicated as other.

| **DQ7 Like** | **DQ8 Like** | **DQ9 Like** | **Other** |
| --- | --- | --- | --- |
| DQB1*03:01:01:01 | DQB1*03:02:01:01 | DQB1*03:03:02:01 | DQB1*03:72 |
| DQB1*03:01:01:02 | DQB1*03:02:01:02 | DQB1*03:03:02:02 |  |
| DQB1*03:01:01:03 | DQB1*03:02:01:03 | DQB1*03:03:02:03 |  |
| DQB1*03:01:01:04 | DQB1*03:02:01:04 | DQB1*03:03:02:04 |  |
| DQB1*03:01:01:05 | DQB1*03:02:01:05 | DQB1*03:03:02:06 |  |
| DQB1*03:01:01:07 | DQB1*03:02:01:06 | DQB1*03:03:02:07 |  |
| DQB1*03:01:01:08 | DQB1*03:02:01:08 | DQB1*03:03:02:09 |  |
| DQB1*03:01:01:10 | DQB1*03:02:01:09 | DQB1*03:03:02:10 |  |
| DQB1*03:01:01:11 | DQB1*03:02:01:10 | DQB1*03:03:02:11 |  |
| DQB1*03:01:01:12 | DQB1*03:02:02:01 | DQB1*03:03:03:01 |  |
| DQB1*03:01:01:14 | DQB1*03:02:02:02 | DQB1*03:03:03:02 |  |
| DQB1*03:01:01:15 | DQB1*03:02:03 | DQB1*03:03:04 |  |
| DQB1*03:01:01:16 | DQB1*03:02:09 | DQB1*03:03:11 |  |
| DQB1*03:01:01:17 | DQB1*03:02:12 | DQB1*03:195 |  |
| DQB1*03:01:01:18 | DQB1*03:02:21 | DQB1*03:239 |  |
| DQB1*03:01:01:19 | DQB1*03:02:22 | DQB1*03:248 |  |
| DQB1*03:01:01:20 | DQB1*03:02:23 | DQB1*03:249 |  |
| DQB1*03:01:01:22 | DQB1*03:02:24 | DQB1*03:25:01 |  |
| DQB1*03:01:01:25 | DQB1*03:02:32 | DQB1*03:31 |  |
| DQB1*03:01:01:26 | DQB1*03:02:37 | DQB1*03:40 |  |
| DQB1*03:01:01:27 | DQB1*03:05:01 | DQB1*03:414 |  |
| DQB1*03:01:01:28 | DQB1*03:05:03 | DQB1*03:453 |  |
| DQB1*03:01:01:29 | DQB1*03:05:04 | DQB1*03:505 |  |
| DQB1*03:01:01:31 | DQB1*03:08 | DQB1*03:507 |  |
| DQB1*03:01:01:32 | DQB1*03:11 |  |  |
| DQB1*03:01:01:33 | DQB1*03:211 |  |  |
| DQB1*03:01:01:34 | DQB1*03:245 |  |  |
| DQB1*03:01:01:35 | DQB1*03:247 |  |  |
| DQB1*03:01:01:39 | DQB1*03:250 |  |  |
| DQB1*03:01:01:42 | DQB1*03:251 |  |  |
| DQB1*03:01:01:43 | DQB1*03:263:01:01 |  |  |
| DQB1*03:01:01:44 | DQB1*03:263:01:02 |  |  |
| DQB1*03:01:01:49 | DQB1*03:289 |  |  |
| DQB1*03:01:01:50 | DQB1*03:37 |  |  |
| DQB1*03:01:01:51 | DQB1*03:415 |  |  |
| DQB1*03:01:01:52 | DQB1*03:442 |  |  |
| DQB1*03:01:02 | DQB1*03:498 |  |  |
| DQB1*03:01:03 | DQB1*03:500 |  |  |
| DQB1*03:01:04:01 | DQB1*03:501 |  |  |
| DQB1*03:01:04:02 | DQB1*03:502 |  |  |
| DQB1*03:01:09 | DQB1*03:504 |  |  |
| DQB1*03:01:17 | DQB1*03:68 |  |  |
| DQB1*03:01:22 |  |  |  |
| DQB1*03:01:35 |  |  |  |
| DQB1*03:01:36 |  |  |  |
| DQB1*03:01:37 |  |  |  |
| DQB1*03:01:41 |  |  |  |
| DQB1*03:01:51 |  |  |  |
| DQB1*03:01:52 |  |  |  |
| DQB1*03:01:53 |  |  |  |
| DQB1*03:01:54 |  |  |  |
| DQB1*03:01:57 |  |  |  |
| DQB1*03:01:58 |  |  |  |
| DQB1*03:04:01:01 |  |  |  |
| DQB1*03:04:01:02 |  |  |  |
| DQB1*03:04:03 |  |  |  |
| DQB1*03:09 |  |  |  |
| DQB1*03:10:02:01 |  |  |  |
| DQB1*03:10:02:02 |  |  |  |
| DQB1*03:114 |  |  |  |
| DQB1*03:115 |  |  |  |
| DQB1*03:12 |  |  |  |
| DQB1*03:150 |  |  |  |
| DQB1*03:187 |  |  |  |
| DQB1*03:19:01:01 |  |  |  |
| DQB1*03:191 |  |  |  |
| DQB1*03:196 |  |  |  |
| DQB1*03:21 |  |  |  |
| DQB1*03:22:01:01 |  |  |  |
| DQB1*03:22:01:02 |  |  |  |
| DQB1*03:243 |  |  |  |
| DQB1*03:246 |  |  |  |
| DQB1*03:252 |  |  |  |
| DQB1*03:253 |  |  |  |
| DQB1*03:254 |  |  |  |
| DQB1*03:29 |  |  |  |
| DQB1*03:309:02 |  |  |  |
| DQB1*03:312 |  |  |  |
| DQB1*03:42 |  |  |  |
| DQB1*03:421 |  |  |  |
| DQB1*03:423 |  |  |  |
| DQB1*03:431 |  |  |  |
| DQB1*03:468 |  |  |  |
| DQB1*03:469 |  |  |  |
| DQB1*03:470 |  |  |  |
| DQB1*03:49:01:01 |  |  |  |
| DQB1*03:49:01:02 |  |  |  |
| DQB1*03:496 |  |  |  |
| DQB1*03:497 |  |  |  |
| DQB1*03:503 |  |  |  |
| DQB1*03:506 |  |  |  |
| DQB1*03:508 |  |  |  |
| DQB1*03:55 |  |  |  |

|  |  |
| --- | --- |

TABLE 1B: Allele names from figure 2 divided in the clusters DQB1*06:01like, DQB1*06:02like, DQB1*06:03like and DQB1*06:04like. The alleles not falling within these clusters are indicated as other.

| **DQB1*06:01 like** | **DQB1*06:02 like** | **DQB1*06:03 like** | **DQB1*06:04 like** | **Other** |
| --- | --- | --- | --- | --- |
| DQB1*06:01:01:01 | DQB1*06:02:01:01 | DQB1*06:03:01:01 | DQB1*06:04:01:01 | DQB1*06:27:01 |
| DQB1*06:01:01:02 | DQB1*06:02:01:02 | DQB1*06:03:01:02 | DQB1*06:04:01:02 | DQB1*06:39 |
| DQB1*06:01:01:03 | DQB1*06:02:01:03 | DQB1*06:03:01:03 | DQB1*06:04:01:03 | DQB1*06:88:01:01 |
| DQB1*06:01:01:04 | DQB1*06:02:01:04 | DQB1*06:03:01:04 | DQB1*06:04:01:04 | DQB1*06:88:01:02 |
| DQB1*06:01:01:05 | DQB1*06:02:01:05 | DQB1*06:03:01:05 | DQB1*06:04:01:05 |  |
| DQB1*06:01:01:06 | DQB1*06:02:01:06 | DQB1*06:03:01:06 | DQB1*06:04:02 |  |
| DQB1*06:01:03 | DQB1*06:02:01:07 | DQB1*06:03:01:07 | DQB1*06:04:03 |  |
| DQB1*06:01:05 | DQB1*06:02:01:08 | DQB1*06:03:01:08 | DQB1*06:04:15 |  |
| DQB1*06:103 | DQB1*06:02:01:09 | DQB1*06:03:01:09 | DQB1*06:04:19 |  |
| DQB1*06:243 | DQB1*06:02:01:10 | DQB1*06:03:01:10 | DQB1*06:04:20 |  |
|  | DQB1*06:02:01:11 | DQB1*06:03:01:11 | DQB1*06:04:21 |  |
|  | DQB1*06:02:01:12 | DQB1*06:03:01:12 | DQB1*06:07:01 |  |
|  | DQB1*06:02:01:13 | DQB1*06:03:01:13 | DQB1*06:07:02 |  |
|  | DQB1*06:02:01:14 | DQB1*06:03:01:14 | DQB1*06:09:01:01 |  |
|  | DQB1*06:02:01:15 | DQB1*06:03:01:15 | DQB1*06:09:01:02 |  |
|  | DQB1*06:02:01:16 | DQB1*06:03:01:16 | DQB1*06:09:01:03 |  |
|  | DQB1*06:02:01:17 | DQB1*06:03:01:17 | DQB1*06:09:02 |  |
|  | DQB1*06:02:01:19 | DQB1*06:03:01:18 | DQB1*06:09:07 |  |
|  | DQB1*06:02:01:20 | DQB1*06:03:01:19 | DQB1*06:118:04 |  |
|  | DQB1*06:02:01:21 | DQB1*06:03:01:20 | DQB1*06:12 |  |
|  | DQB1*06:02:01:22 | DQB1*06:03:01:21 | DQB1*06:164 |  |
|  | DQB1*06:02:01:23 | DQB1*06:03:02 | DQB1*06:171 |  |
|  | DQB1*06:02:01:24 | DQB1*06:03:11 | DQB1*06:206:02 |  |
|  | DQB1*06:02:01:25 | DQB1*06:03:12 | DQB1*06:21 |  |
|  | DQB1*06:02:01:26 | DQB1*06:03:14 | DQB1*06:217 |  |
|  | DQB1*06:02:01:27 | DQB1*06:03:21 | DQB1*06:275 |  |
|  | DQB1*06:02:01:28 | DQB1*06:03:23 | DQB1*06:34 |  |
|  | DQB1*06:02:01:29 | DQB1*06:03:24 | DQB1*06:353 |  |
|  | DQB1*06:02:01:30 | DQB1*06:03:25 | DQB1*06:358 |  |
|  | DQB1*06:02:01:31 | DQB1*06:03:26 | DQB1*06:36 |  |
|  | DQB1*06:02:02 | DQB1*06:03:41 | DQB1*06:38 |  |
|  | DQB1*06:02:07 | DQB1*06:03:42 | DQB1*06:381 |  |
|  | DQB1*06:02:17 | DQB1*06:03:43 | DQB1*06:398 |  |
|  | DQB1*06:02:22 | DQB1*06:03:45 | DQB1*06:407 |  |
|  | DQB1*06:02:23 | DQB1*06:08:01 | DQB1*06:41:01:01 |  |
|  | DQB1*06:02:26 | DQB1*06:110 | DQB1*06:41:01:02 |  |
|  | DQB1*06:02:27 | DQB1*06:145:02 | DQB1*06:41:01:03 |  |
|  | DQB1*06:02:28 | DQB1*06:185 | DQB1*06:429 |  |
|  | DQB1*06:02:40 | DQB1*06:187 | DQB1*06:444 |  |
|  | DQB1*06:02:44 | DQB1*06:218 | DQB1*06:449 |  |
|  | DQB1*06:02:50 | DQB1*06:221 | DQB1*06:452N |  |
|  | DQB1*06:02:53 | DQB1*06:222 | DQB1*06:458N |  |
|  | DQB1*06:02:54 | DQB1*06:223 | DQB1*06:84:01:01 |  |
|  | DQB1*06:02:55 | DQB1*06:28 | DQB1*06:84:01:02 |  |
|  | DQB1*06:02:56 | DQB1*06:362 |  |  |
|  | DQB1*06:02:57 | DQB1*06:365 |  |  |
|  | DQB1*06:02:59 | DQB1*06:391 |  |  |
|  | DQB1*06:10 | DQB1*06:396 |  |  |
|  | DQB1*06:109 | DQB1*06:399 |  |  |
|  | DQB1*06:11:01:01 | DQB1*06:400 |  |  |
|  | DQB1*06:11:01:02 | DQB1*06:403 |  |  |
|  | DQB1*06:111 | DQB1*06:410 |  |  |
|  | DQB1*06:117 | DQB1*06:424 |  |  |
|  | DQB1*06:127 | DQB1*06:433 |  |  |
|  | DQB1*06:13:01 | DQB1*06:44 |  |  |
|  | DQB1*06:15:02 | DQB1*06:450 |  |  |
|  | DQB1*06:16 | DQB1*06:453 |  |  |
|  | DQB1*06:188 | DQB1*06:454N |  |  |
|  | DQB1*06:20 | DQB1*06:455 |  |  |
|  | DQB1*06:219 | DQB1*06:459 |  |  |
|  | DQB1*06:224 | DQB1*06:460 |  |  |
|  | DQB1*06:225 | DQB1*06:90 |  |  |
|  | DQB1*06:226 |  |  |  |
|  | DQB1*06:227 |  |  |  |
|  | DQB1*06:228 |  |  |  |
|  | DQB1*06:284 |  |  |  |
|  | DQB1*06:286 |  |  |  |
|  | DQB1*06:293 |  |  |  |
|  | DQB1*06:298 |  |  |  |
|  | DQB1*06:306N |  |  |  |
|  | DQB1*06:317N |  |  |  |
|  | DQB1*06:33 |  |  |  |
|  | DQB1*06:37 |  |  |  |
|  | DQB1*06:383N |  |  |  |
|  | DQB1*06:384 |  |  |  |
|  | DQB1*06:395 |  |  |  |
|  | DQB1*06:397N |  |  |  |
|  | DQB1*06:401 |  |  |  |
|  | DQB1*06:402 |  |  |  |
|  | DQB1*06:404 |  |  |  |
|  | DQB1*06:405 |  |  |  |
|  | DQB1*06:406 |  |  |  |
|  | DQB1*06:408 |  |  |  |
|  | DQB1*06:409 |  |  |  |
|  | DQB1*06:411 |  |  |  |
|  | DQB1*06:412 |  |  |  |
|  | DQB1*06:413 |  |  |  |
|  | DQB1*06:417 |  |  |  |
|  | DQB1*06:422N |  |  |  |
|  | DQB1*06:442 |  |  |  |
|  | DQB1*06:448 |  |  |  |
|  | DQB1*06:451 |  |  |  |
|  | DQB1*06:456N |  |  |  |
|  | DQB1*06:457 |  |  |  |
|  | DQB1*06:46 |  |  |  |
|  | DQB1*06:461 |  |  |  |
|  | DQB1*06:48:01 |  |  |  |
|  | DQB1*06:49 |  |  |  |
|  | DQB1*06:73 |  |  |  |
|  | DQB1*06:74 |  |  |  |
|  | DQB1*06:75N |  |  |  |

TABLE 1C: Allele names from figure 3 divided in the clusters B*18:01:01:01like and B*18:01:01:02like. The alleles not falling within these clusters are indicated as other.

| **B*18:01:01:01 like** | **B*18:01:01:02 like** | **Other** |
| --- | --- | --- |
| B*18:01:01:01 | B*18:01:01:02 | B*18:01:01:18 |
| B*18:01:01:06 | B*18:01:01:05 | B*18:03:01:02 |
| B*18:01:01:08 | B*18:01:01:11 |  |
| B*18:01:01:15 | B*18:01:01:12Q |  |
| B*18:01:01:19 | B*18:01:01:14 |  |
| B*18:01:01:30 | B*18:01:01:17 |  |
| B*18:01:01:51 | B*18:01:01:20 |  |
| B*18:01:43 | B*18:01:01:22 |  |
| B*18:07:03:01 | B*18:01:01:23 |  |
| B*18:12:01 | B*18:01:01:52 |  |
| B*18:156:01:01 | B*18:01:01:61 |  |
| B*18:197 | B*18:01:01:74 |  |
| B*18:226 | B*18:01:06 |  |
|  | B*18:01:32 |  |
|  | B*18:01:34 |  |
|  | B*18:01:35 |  |
|  | B*18:01:50 |  |
|  | B*18:02:01 |  |
|  | B*18:04:01 |  |
|  | B*18:14 |  |
|  | B*18:143 |  |
|  | B*18:144 |  |
|  | B*18:156:01:02 |  |
|  | B*18:157:01:01 |  |
|  | B*18:166 |  |
|  | B*18:170 |  |
|  | B*18:171 |  |
|  | B*18:172 |  |
|  | B*18:173 |  |
|  | B*18:174 |  |
|  | B*18:175 |  |
|  | B*18:18:01:02 |  |
|  | B*18:194:01:02 |  |
|  | B*18:196 |  |
|  | B*18:198 |  |
|  | B*18:199 |  |
|  | B*18:202 |  |
|  | B*18:213 |  |
|  | B*18:214 |  |
|  | B*18:215 |  |
|  | B*18:216 |  |
|  | B*18:217 |  |
|  | B*18:34 |  |
|  | B*18:37:02 |  |
